# Effects of Implant Surface Debridement and Systemic Antibiotics on the Clinical and Microbiological Variables of Periimplantitis

**DOI:** 10.1155/2021/6660052

**Published:** 2021-01-22

**Authors:** Muhammad Irshad, Mohammad Khursheed Alam, Sajid Ali, Ahmad Alawneh, Mohammed Alhadi, Ahmed Alhadi, Ahmed Ali Alfawzan

**Affiliations:** ^1^Department of Oral Pathology, Rehman College of Dentistry, Peshawar, Pakistan; ^2^Department of Preventive Dentistry, College of Dentistry, Jouf University, Sakaka 72345, Saudi Arabia; ^3^Department of Prosthodontics, Rehman College of Dentistry, Peshawar, Pakistan; ^4^Jordanian Royal Medical Services, Dental Department, Jordan; ^5^Aljouf Specialist Dental Center, MOH, Sakaka 72345, Saudi Arabia; ^6^Ministry of Health in Saudi Arabia, Saudi Arabia; ^7^Department of Preventive Dentistry, College of Dentistry in Ar Rass, Qassim University, Ar Rass, Saudi Arabia

## Abstract

**Objective:**

To investigate the role of implant surface debridement alone and in conjunction with systemic antibiotics on the clinical and microbiological variables of periimplantitis.

**Materials and Methods:**

Data of forty-six patients with at least one dental implant having bleeding-on-probing (BoP), probing pocket depth (PPD) of more than 5 mm, and radiographic bone loss of more than 3 mm were retrieved from clinical records. Data was recorded for dental implant with the deepest PPD, BoP, and bone loss from each patient. “Group-A” received implant surface debridement alone, while “group-B” additionally received systemic antibiotics. Clinical and microbiological data of patients were compared before and after the treatment.

**Results:**

At the implant level, a significant reduction of PPD, mucosal recession (MR), and BoP was achieved for all patients. Group B achieved significant improvement in MR and BoP compared to group A at implant level. PPD, MR, and plaque scores showed improvement at implant site level. At 3 months recall visit, 44% of group A and 52% of group B implants required surgical treatment. The presence and proportions of studied bacteria of both groups did not differ significantly at the recall visit when compared to the initial visit. However, P. intermedia and P. micros showed a significant reduction in group A at the recall visit.

**Conclusions:**

Implant surface debridement improved the clinical parameters of periimplantitis. In addition, adjunctive use of systemic antibiotics increased mucosal recession and improved bleeding on probing in periimplantitis.

## 1. Introduction

Periimplantitis is a chronic, inflammatory disease characterized by gradual breakdown of the soft and hard tissues around a dental implant [1, 2]. Without proper management, periimplantitis can cause mobility and eventual loss of the affected dental implant. Periimplantitis may affect 6.6%-34% of all the dental implants over a period of 14 years [3, 4].

The etiology of periimplantitis is multifactorial in nature; however, bacteria play a vital role in disease initiation and progression [5]. Significant differences have been reported in the microbiota associated with diseased implants compared to healthy dental implants [6, 7]. In contrast to healthy implants which mainly have a biofilm composed of Gram-positive cocci [6, 8], the biofilm associated with periimplantitis is characterized by the predominance of anaerobic bacteria. Prevotella intermedia/nigrescens, Porphyromonas gingivalis, and Aggregatibacter actinomycetemcomitans are some of the most common bacteria associated with periimplantitis [9, 10]. Multiple similarities could be drawn between periimplantitis and periodontitis including similar bacterial species associated with both diseases [9]. However, microbial species unique to periimplantitis have also been reported in the literature [6, 11–16].

Studies on the treatment outcome of periimplantitis are scarce, and evidence of a single effective treatment modality for periimplantitis is inconclusive [17]. Antibiotics along with implant surface cleaning/debridement ha**ve** been reported to improve the clinical and microbiological parameters in periimplantitis [18]. The effects of adjunctive antibiotic treatment remained significant even after one-year posttreatment when compared to baseline. This study did not have a control group which makes the utility of adjunctive antibiotics use uncertain in the treatment of periimplantitis. As far as we know, the role of systemic antibiotics in addition to implant surface debridement has not been studied before. Therefore, we aimed to investigate the role of implant surface debridement alone and in conjunction with systemic antibiotics on the clinical and bacteriological variables of periimplantitis.

## 2. Materials and Methods

In this retrospective study, the patient database of the Ace Dental and Implant Center (a privately-owned clinic in Peshawar, Pakistan) was searched for periimplantitis patients based on the following criteria as suggested by Renvert et al. [19].

1. Bleeding/suppuration on probing (BoP)
2. Probing pocket depths (PPD) of more than 5 mm
3. Radiographic bone loss of more than 3 mm (periapical radiographs were used to measure bone loss from the first implant thread to crestal bone)

The inclusion criteria were as follows:

1. Patients with minimum one titanium dental implant diagnosed with periimplantitis
2. Dental implants must be in use for minimum period of 1 year or more and
3. Patients older than 18 years

Patients were excluded from the study if:

1. Systemic antibiotics were used in the 3 months before treatment or
2. Nonsteroidal anti-inflammatory drugs were used in the past four weeks
3. Patients with diabetes and other chronic systemic disease were also excluded

Sample size was calculated using G∗ Power software version 3.1.9.4 at an effect size of 0.39, alpha = 0.05, and power of the study = 0.80. A total of 46 patient records were obtained from the database.

### 2.1. Data Collection

The following data was obtained at the initial visit (before starting the treatment): (1) age (in years), (2) sex, (3) presence of chronic systemic disease [3, 20], (4) dental status (edentulous, dentate, and number of remaining teeth), (6) present or past smoking history, and (7) history of periodontitis. Moreover, implants having the greatest probing measurements were selected as target implants, and while deepest pockets were selected as target implant sites.

The following clinical measurements were obtained for all teeth/implants present at the initial and at the recall visits (3 months after the initial visit): (1) plaque scores (measured by the modified plaque index proposed by Van der Weijden et al. [21]), (2) bleeding/suppuration on probing, (3) PPD in mm, (4) clinical attachment level (CAL), and (6) mucosal recession was calculated by subtracting PPD from CAL (MR = CAL − PPD). A single operator made all the measurements around the target dental implant using a Marquis CP-12 probe (Hu-Friedy, Chicago, Illinois, USA).

Data on the use and type of systemic antibiotics during periimplantitis treatment was retrieved from the patient database. Data of patients who received a standard antibiotic regimen (amoxicillin 500 mg three times a day plus metronidazole 400 mg twice a day for 5 days) was selected for the study. Submucosal plaque samples had been previously obtained by using sterile paper points from the deepest implant sites at the initial visit as well as 3 months recall visit. Microbiological data was obtained from the laboratory records.

Data was made fully anonymous by assigning a serial number to each record, and ethical approval (EC Ref. No. RCD-19-04-018) was obtained from the institutional ethical committee of Rehman College of Dentistry, Peshawar.

### 2.2. Initial Visit

Data of all periimplantitis patients referred to the Ace Dental and Implant Center, University Town Peshawar, for treatment of periimplant infection was evaluated. Past medical and dental histories were recorded at the initial visit. Patients were divided into two groups, group A (*n* = 25) who had received implant surface debridement along with a standard regimen of antibiotics (amoxicillin 500 mg three times a day plus metronidazole 400 mg twice a day for 5 days), while group B included patients who only received implant surface debridement without the use of systemic antibiotics.

### 2.3. Microbiological Analysis

Sterile paperpoints were used to obtain submucosal plaque from the periimplant pocket with the greatest PPD measurement [22]. Subsequently, paperpoints were transferred to 5 ml sterile tubes with standard reduced transport fluid (a ditheithreitol poised mineral salt solution) [23]. Within 2 hours of collection, all samples were carried to the Veterinary Research Institute (VRI), Peshawar, for microbiological culture.

Selected bacterial species were anaerobically cultured according to the standard methods [24] Serial dilutions of the previously obtained submucosal plaque samples were cultured on 5% horse blood agar plates (Oxoid no.2, Basingstoke, UK) supplemented with hemin (5 mg/l) and menadione (1 mg/l). Trypticase soy-serum-bacitracin-vancomycin (TSBV) plates were used as culture medium for the A. actinomycetemcomitans growth. Incubation of blood agar culture plates was carried out in an anaerobic environment (80%N2, 10%H2, and at 10%CO2) at a temperature of 37°C. TSBV plates were incubated and were carried out at 5%CO2 for up to two weeks. Bacterial colonies were counted three times on agar plates using a magnifying glass, and the average was taken to calculate colony forming units per ml (CFU/ml). The presence and relative proportions of target bacteria were noted. Colony morphology, Gram-staining & microscopy, anaerobic growth, fermentation of glucose, and indole were used to identify bacterial species.

### 2.4. Implant Surface Debridement

Before commencement of the nonsurgical treatment, patients were provided a commercially available 0.12% chlorhexidine mouthwash to rinse for one minute. Local anesthesia was administered to the affected implant (medicaine 2%, 1 : 100000 epinephrine), and debridement of implant surface was carried out with an ultrasonic scaler having specialized tip for implant surface (WoodPecker; Guilin Zhuomuniao Medical Devices, Guilin, China). Patients having gingivitis or periodontitis were also treated. A generic mouthwash containing 0.12% chlorhexidine was prescribed, and patients were instructed to use it three times a day for 30 days [25]. Standard oral hygiene instructions (OHI) were given to all patients.

### 2.5. Recall Visit

After 3 months of the initial visit, patients were again examined by the same clinician (MI), and clinical measurements were recorded. Patients were referred for periimplant surgery if indicated.

### 2.6. Statistical Analysis

GraphPad Prism software (version 5.00 for Windows, San Diego California, USA) was used for data analysis. To compare continuous and categorical variables, Wilcoxon signed ranks and McNemar tests were used, respectively. Differences were considered significant at a *p* value of ≤5.

## 3. Results

Table 1 presents general features of patients included in the study. Forty-six (46) patients, ages ranging from 42 to 71 years (55.7 ± 15), were included in the study. The participants comprised of 34 males and 12 females. Group A included 25 patients who received a standard regimen of systemic antibiotics as mentioned earlier in the materials and methods part, while 21 patients received implant surface debridement alone.

### 3.1. Clinical Parameters at the Target Implant Site

Table 2(a) presents a comparison of the clinical parameters between initial and recall visits. The studied clinical parameters did not differ significantly between the two groups at the initial visit. PPD of the target sites decreased significantly (*p* < 0.001) in both groups at recall visit in comparison to the initial visit. The mean PPD of group B was significantly lower than group A (*p* = 0.003), when both groups were compared at the recall visit. Measurements of the CAL significant changed only in group B (*p* = 0.002), while it was not significant in group A (*p* = 0.12). For both groups, values of MR were significantly higher at the recall visit in comparison to the initial visit (group B, *p* = 0.002; group A, *p* = 0.01). In addition, the mean MR values were significantly greater in group A (*p* = 0.005) in comparison to group B at the recall visit. Significant reduction of BoP was also observed for both the groups at the recall visit compared to the initial visit (group B, *p* = 0.03; group A, *p* = 0.011). The deepest periimplant pockets showed the greatest PPD and MR changes in both groups.

Plaque scores and suppuration on probing did not change significantly for both groups at the recall visit.

### 3.2. Clinical Parameters of the Target Implants

Both groups showed significantly lower PPD values around the target implants at the recall visit (group B: *p* = 0.003 and group A: *p* = 0.04) when compared to the initial visit (Table 2(b)). CAL showed no significant change for both groups at the recall visit when compared to the initial visit. Only group B showed a significant increase in MR in comparison with the initial visit (*p* = 0.001) and in comparison, with group A (*p* = 0.012). Moreover, BoP in the group B showed a significant change at recall visit when compared to; initial visit (*p* = 0.001) and to group A (*p* = 0.02). Suppuration on probing showed no significant change, while plaque scores decreased significantly when compared to the initial visit for both groups (group B: *p* = 0.04 and group A: *p* = 0.01). In group B, 44% of patients needed surgery, while in group A, 52% of the target implants were referred for periimplant surgery at the recall visit.

### 3.3. Microbiological Parameters

Table 3 presents microbiological data of the implants. Differences between the mean proportions and prevalence of studied bacterial species of the two groups at the initial visit were not significant. Similarly, group A did not show significant changes in the prevalence or proportions of the bacterial species between initial and recall visits. Interestingly, the prevalence of P. intermedia and P. micros in group A was significantly lower at recall visit (*p* = 0.002 and *p* = 0.001, respectively) compared to the initial visit. Moreover, a reduction in proportions of P. intermedia was observed (*p* = 0.04) in the group A at the recall visit.

No significant differences could be detected in the bacterial loads (average CFU/ml) of the two groups at the target implant level. The growth of A. actinomycetemcomitans could not be confirmed in any of the patient samples.

## 4. Discussion

The present study evaluated the effects of adjunctive systemic antibiotics and implant surface debridement on the clinical and microbiological parameters of periimplantitis. The use of antibiotics improved the mean PPD, MR, and BoP in periimplantitis. Moreover, significant improvements were observed in PPD and MR with implant surface debridement combined with systemic antibiotics at the implant sites with the greatest PPD measurements, and MR and BoP at implant level in comparison to implant surface debridement only.

Limited studies are available on the effectiveness of implant surface debridement alone and in combination with systemic antibiotics; therefore, more research is needed to elucidate its role in the evidence-based management of periimplantitis [26]. One uncontrolled cohort study has reported improvement in the clinical parameters of periimplantitis with implant surface debridement combined with systemic antibiotics [18]. A literature review including 16 studies has suggested that nonsurgical treatment alone has no or minimal effects on improving the clinical parameters of periimplantitis [27]. However, they observed improvement in BoP and PPD with mechanical debridement combined with systemic antibiotics. These findings are in line with the current study.

Another literature review has reported that nonsurgical treatment/implant surface debridement has limited or no value in periimplantitis treatment and all affected implants invariably need surgical treatment over a period of time [28]. In contrast, we found that more than 50% of patients did not need surgery at the recall visit regardless of antibiotics use. Since we only followed the patients for three months, the proportions of patients requiring surgical treatment might increase with a longer follow-up time.

The absence of pus has been previously suggested to correlate with the success of periimplantitis treatment [29]. Implants having pus at the initial visit consistently needed surgical management after three months of debridement as described by Thierbach et al. [29], while those showing no pus on probing at the beginning did not need surgery. This finding could not be verified in our results.

Moreover, *P. gingivalis* was completely eradicated in group B (with antibiotics) at the recall visit in contrast to group A (no antibiotics) where the prevalence and proportions of *P. gingivalis* were unaffected. Previous reports suggest that the combined effects of amoxicillin and metronidazole are effective against *P. gingivalis*, which substantiates our findings [24]. Intriguingly, a lower frequency of *P. intermedia* and *P. micros* was found only in group A. Effectiveness of implant surface debridement alone in decreasing the prevalence and proportions of *P. intermedia* and *P. micros* in periodontal disease has been previously reported [30].

Multiple aspects of periimplantitis are like chronic periodontitis, both are opportunistic infections, triggered by the existence of bacteria and an aberrant response from the host immune system [2]. Due to the close similarities, periimplantitis is usually treated in a similar manner to periodontitis, consisting of mechanical debridement and use of local and systemic antibacterial agents [2]. Recent studies, however, suggest that important differences could exist between the microbiota associated with periimplantitis compared to periodontitis [31, 32]. Large-scale microbiological studies using open-ended microbial detection techniques are required to further elucidate the role of specific microbial species in the etiology and pathogenesis of periimplantitis. In addition, the behavior of biofilm on implant surface and its interaction with host immune system in the presence of implant biomaterial also need further investigation [33].

## 5. Conclusions

In the current study, adjunct use of systemic antibiotics did not demonstrate an additional advantage in reducing periimplant bacterial species and total bacterial loads. Implant surface debridement alone is effective in improving the clinical parameters of periimplantitis. In addition, adjunctive use of systemic antibiotics significantly reduced pocket probing depths, increased mucosal recession, and decreased bleeding on probing in periimplantitis.

## Figures and Tables

**Table 1:** Characteristics of the patients at the initial visit (*N* = 46).

Age (mean ± SD)	42-71 (55.7 ± 15)	
Gender	Male	34 (74)
	Female	12 (26)
Dental status (*N*, %)	Edentulous	14 (30)
	Dentate	32 (70)
Smoking habits	Smoker	4 (8)
	Nonsmoker	31 (67)
	Past-smoker	5 (11)
	Not known	6 (13)
Past history of periodontitis	Yes	17 (37)
	No	24 (52)
	Unknown	5 (11)

**Table 2:** Clinical measurements of target implant site (a) and target implant (b) at initial visit and three-month recall visit of patients group A (with antibiotics, *N* = 25) and group B patients (without antibiotics, *N* = 21).

	Initial visit	Recall visit	*p* value initial vs. recall visit	*p* value evaluation group B vs. group A
A. Target implant site				
PPD (mm ± SD)				
Total	7.3 (1.7)	5.3 (1.4)	<0.001	
Group B	7.5(1.6)	4.6 (1.2)	<0.001	
Group A	7.6 (1.4)	5.2 (1.3)	<0.001	0.003
CAL (mm ± SD)				
Total	11.2 (2.0)	10.3 (1.4)	0.001	
Group B	12.0 (1.8)	10.4 (1.6)	0.003	
Group A	11.0 (1.7)	10.6 (1.7)	0.12	0.3
MR (mm ± SD)				
Total	4.3 (1.9)	5.2 (2.2)	0.001	
Group B	4.5 (2.0)	6.3 (1.6)	0.002	
Group A	3.8 (1.4)	4.5 (2.3)	0.01	0.005
BoP (%)				
Total	100	84	0.004	
Group B	100	86	0.03	
Group A	100	78	0.011	0.4
Suppuration on probing (%)				
Total	23	9	0.20	
Group B	27	8	0.09	
Group A	19	8	0.33	0.2
Plaque scores (%)				
Total	33	24	0.2	
Group B	36	38	0.59	
Group A	30	10	0.07	0.1
B. Target implant				
PPD (mm ± SEM)				
Total	5.6 (1.2)	4.7 (1.1)	<0.001	
Group B	5.4 (0.9)	4.3 (0.6)	0.003	
Group A	5.5 (1.5)	4.8 (1.3)	0.04	0.07
CAL (mm ± SEM)				
Total	11.1 (2.2)	10.3 (2.2)	0.34	
Group B	12.1 (2.3)	10.7 (2.0)	0.2	
Group A	9.6 (2.1)	9.9 (2.2)	0.6	0.32
MR (mm ± SEM)				
Total group	4.8 (2.1)	5.4 (2.5)	0.001	
Group B	5.9 (2.1)	6.6 (1.8)	0.001	
Group A	4.6 (1.6)	4.7 (2.3)	0.17	0.012
BoP				
Total	5.1 (1.2)	3.7 (1.8)	<0.001	
Group B	5.2 (1.1)	3.0 (1.9)	0.001	
Group A	5.0 (1.4)	4.1 (1.6)	0.08	0.02
Suppuration on probing				
Total group	1.0 (1.6)	0.3 (1.2)	0.05	
Group B	0.8 (1.1)	0.4 (1.4)	0.23	
Group A	0.9 (1.9)	0.3 (1.1)	0.17	0.8
Plaque scores				
Total	2.6 (2.3)	0.8 (1.3)	0.01	
Group B	2.4 (2.1)	1.2 (1.5)	0.04	
Group A	2.7 (2.5)	0. (0.7)	0.01	0.1

Group B: implant surface debridement alone; Group A: implant surface debridement with adjunctive systemic antibiotics.

**Table 3:** Prevalence and proportions (±SD) of the studied bacteria at the target implant site as at initial and recall visits (*N* = 46).

Bacterial species								
		Group B (*N* = 25)			Group A (*N* = 21)		Recall visit group B vs. group A	
		Initial visit	Recall visit		Initial visit	Recall visit		
*A. actinomycetemcomitans*	Prevalence *N* (%) Mean (±SD) proportion	0 (0) 0 (0)	0 (0) 0 (0)	ns^†^ ns	0 (0) 0 (0)	0 (0) 0 (0)	ns ns	ns ns
*P. gingivalis*	Prevalence *N* (%) Mean (±SD) proportion	4 (16) 1.6 (4.3)	0 (0) 0 (0)	ns ns	6 (28.5) 5.3 (14.5)	5 (24) 36.1(17.5)	ns ns	0.06 ns
*P. intermedia*	Prevalence *N* (%) Mean (±SD) proportion	6 (24) 1.8 (3.2)	2 (8) 3.8 (2.8)	ns ns	8 (38) 2.6 (4.1)	4 (19) 1.5 (2.3)	0.002 0.04	ns ns
*T. forsythia*	Prevalence *N* (%) Mean (±SD) proportion	9 (36) 2.3 (4.4)	6 (24) 3.6 (5.1)	ns ns	7 (33) 1.4 (3.4)	5 (24) 3.6 (3.4)	ns ns	ns ns
*P. micros*	Prevalence *N* (%) Mean (±SD) proportion	18 (72) 19.2 (22.4)	14 (56) 12.1 (14.3)	ns ns	17 (81) 19.8 (24.1)	10 (47.6) 8.2 (9.5)	0.001 ns	ns ns
*F. nucleatum*	Prevalence *N* (%) Mean (±SD) proportion	15 (60) 3.1 (5.3)	14 (56) 3.4 (6.3)	ns ns	15 (71) 2.1 (7.2)	13 (62) 1.9 (4.5)	ns ns	ns ns
*C. rectus*	Prevalence *N* (%) Mean (±SD) proportion	2 (8) 4.4 (2.2)	2 (8) 2.28 (0)	ns ns	1 (4.8) 2.0 (0)	1 (4.8) 1.5 (2.6)	ns ns	ns ns
Total CFU count		5.4 × 10^6^ (5.9 × 10^6^)		ns	3.8 × 10^6^ (2.8 × 10^6^)		ns	

Group B: implant surface debridement alone; Group A: implant surface debridement with adjunctive systemic antibiotics; †ns: not significant.

## Data Availability

Details are presented within the article in the form of tables and text in results. Other data will be made available upon request.
